# Effects of Hydroxylpropyl-β-Cyclodextrin on *in Vitro* Insulin Stability

**DOI:** 10.3390/ijms10052031

**Published:** 2009-05-06

**Authors:** Liefeng Zhang, Wenjie Zhu, Lingling Song, Yifan Wang, Hui Jiang, Suyun Xian, Yong Ren

**Affiliations:** Jiangsu Key Laboratory for Supramolecular Medicinal and Applications, Nanjing Normal University, Nanjing 210097, P.R. China; E-Mails: lfzhang1010@yahoo.com.cn (L.-F.Z.); ersaclarke@gmail.com (W.-J.Z.)

**Keywords:** Protection, insulin, stability, cyclodextrin

## Abstract

The objective of this study was to elucidate the effects of hydroxylpropyl-β-cyclodextrin (HP-β-CD) on the *in vitro* stability of insulin. It was found that HP-β-CD had positive effects on the stability of insulin in acid and base and under high temperature conditions. Furthermore, use of HP-β-CD could also increase the stability of disulfide bonds which are important to the conformation of insulin. Through ^1^H-NMR experiments it was found that the protective effect of HP-β-CD was due to complexation with insulin. The results suggest that the presence of HP-β-CD could improve the stability of insulin in different environments.

## 1. Introduction

Insulin is a hormone produced in the pancreas to decrease the level of sugar in the blood and it was the first protein to be sequenced, in 1955, by Frederick Sanger [1,2]. Insulin is the most effective drug in diabetes treatment. The insulin molecule consists of 51 amino acid residues and three disulfide bonds. Insulin is made up of two peptide chains, A (with 21 amino acid residues) and B (with 30 residues), linked by two disulfide bonds connecting cysteine residues A7 and B7 and connecting residues A20 and B19. The A chain has an additional internal disulfide bond between residues A6 and A11. The stability of disulfide bonds is important to the conformation of insulin. The stability of insulin under different conditions has been studied by many researchers. Most studies have dealt with the hydrolysis in acid medium into deamidoinsulin products [3]. Decomposition of insulin during storage of bovine insulin in the solid state had been studied by Fisher and Porter [4]. Chemical stability of insulin was studied by Brange *et al.* [5]. In a word, the stability of insulin in pharmaceutical preparations needs to be improved.

Cyclodextrins (CDs) are cyclic (α-1, 4)-linked oligosaccharides with 6-, 7- or 8-α-d-glucopyranose, units containing a relatively hydrophobic central cavity and a hydrophilic outer surface [6,7]. β-cyclodextrin (β-CD) consists of seven glucosides. β-CD and its derivatives are widely used as solubilizers, stabilizers, absorption promoters, and excipients. 2-Hydroxypropyl-β-cyclodextrin (HP-β-CD) is a common β-CD derivative with improved water solubility properties [8] and which is also more toxicologically benign [9]. It is believed that β-CD can form non-covalent inclusion complexes with a wide variety of drugs/proteins. The complexation often alters the physic-chemical and biological properties of guest molecules [10]. Hydrophilic β-CD inhibits the adsorption of insulin to hydrophobic surfaces and prevents self-aggregating nature of insulin at neutral pH [11,12]. Dotsikas and Loukas [13] reported that methyl-β-CD, another β-CD derivative, had a pronounced stabilizing effect on insulin decomposition in high temperature. Cyclodextrin (CD) complexation represents a unique and effective strategy for improving protein therapy by stabilizing them against aggregation [11], thermal denaturation and degradation. In our laboratory it was found that HP-β-CD, casein and protamine could offer some positive and useful results, and could protect insulin from degradation during transit through the intestinal tract [15]. However, different results were obtained under different conditions. Shao *et al.* [14] reported that hydroxypropyl-β-cyclodextrin did neither increase oral bioavailability of insulin, nor it could protect insulin from α-chymotrypsin degradation. Taking this information into account, HP-β-CD was chosen to study its effect on insulin under different physical and chemical conditions. The investigation was focused on whether acid and base solutions or high temperature affected the stability of insulin, and whether HP-β-CD affected it. Moreover, whether HP-β-CD affected the stability of disulfide bonds or not was investigated. In addition, the complexation of insulin with HP-β-CD was characterized in aqueous media by ^1^H-NMR chemical shift displacements under the addition of HP-β-CD.

## 2. Results and Discussion

### 2.1. Effect of HP-β-CD on insulin in acid solution

**Figure 1.**
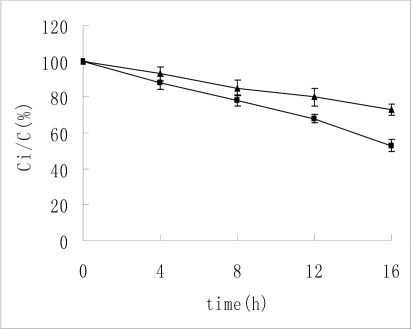
The remaining insulin in acid solution. ▴-▴ indicates the concentration change of insulin without HP-β-CD. ▪—▪ indicates the concentration change of insulin with the existence of HP-β-CD. n = 3, mean ± SD.

Most previous studies had dealt with insulin hydrolysis in acid media into deamido products. Deamidation takes place at position 21 in the A-chain of insulin (Asn(Asparagine)A21) [3,4]. In an acid environment (pH ≤ 2), insulin molecules are in monomer state [16]. This makes them very unstable. The amount of insulin in acid solution was determined by HPLC. The results are presented in Table 1 and Figure 1. After incubation in pH 1 HCl solution for 16 hours, only 53% of the original amount of insulin remained. But about 73% of the insulin remained undegraded when it was treated with HP-β-CD. It indicated that HP-β-CD could partially protect insulin from acid degradation. The percentage data in Figure 1 (as in the figures that follow) was calculated by comparing the peak areas after and before incubation for different time intervals.

### 2.2. Effect of HP-β-CD on insulin in base solution

In a basic environment (pH > 9), insulin will disassociate to monomer, and deactivate due to the structural change. The concentration of insulin in base solution is determined in the similar way as in acid solution.

**Figure 2.**
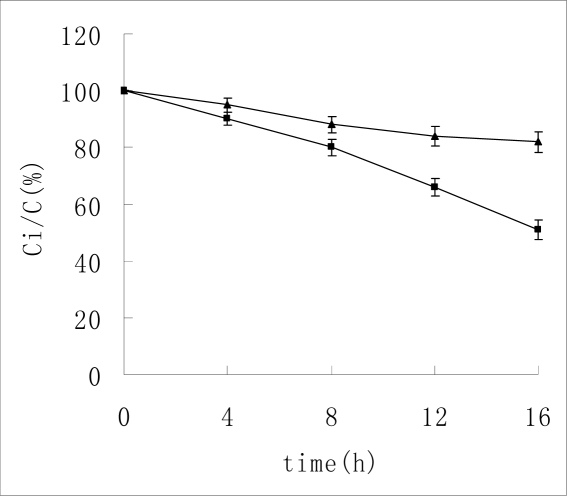
The remaining insulin in base solution. ▴-▴ indicates the concentration change of insulin without HP-β-CD. ▪—▪ indicates the concentration change of insulin with the existence of HP-β-CD. n = 3, mean ± SD.

As shown in Figure 2, about 51% of the original amount remained after incubation in pH 13 NaOH solution for 16 hours. But 82% remained in HP-β-CD/Insulin complex solution. It indicated that HP-β-CD could also partially protect insulin from degradation in basic solution. What is more, the complex between insulin and HP-β-CD was more stable in base solution than in acid solution, which could be due to the fact that HP-β-CD was not stable in acid solution.

### 2.3. Effect of HP-β-CD on insulin in high temperature

**Figure 3.**
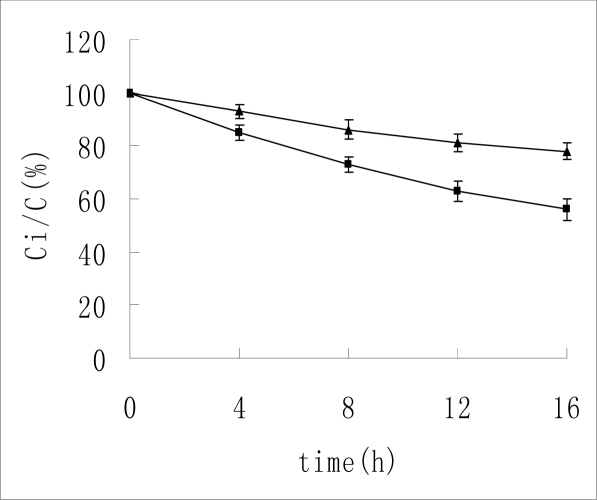
The remaining insulin in a 55 °C water bath. ▴-▴ indicates the concentration change of insulin without HP-β-CD. ▪—▪ indicates the concentration change of insulin with the existence of HP-β-CD. N = 3, mean ± SD.

Insulin is unstable in high temperature. Huus *et al.* [17] reported deamidation of Asn (B3) was observed in high temperature environments. The stability of insulin at 55 °C was investigated. The results are presented in the Figure 3. As shown in the table and figure, after placement in a 55 °C water bath, only 61% insulin remained without HP-β-CD. But 79% insulin remained in HP-β-CD/insulin complex solution. Obviously, HP-β-CD helped insulin to resist heat. This result was consistent with the report by Dotsikas *et al.* [13], although they used methyl-β-CD.

### 2.4. Effect of HP-β-CD on the stability of disulfide bonds

**Figure 4.**
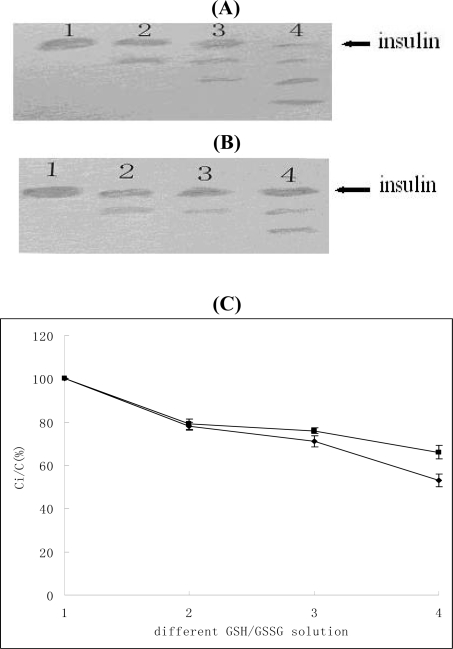
Effect of HP-β-CD on the stability of disulfide bonds. A and B are the PAGE results. The upper graph (A) was insulin without HP-β-CD, the lower graph (B) was insulin and HP-β-CD. Lanes 1-4 in (A) and (B) indicated insulin incubated with different GSH/GSSG (mM/mM) solutions with 0, 20/1, 40/1, 60/1, GSH/GSSG solution, respectively. C is the result of HPLC. ▪—▪ indicates the remaining insulin with HP-β-CD, ♦-♦ indicates the remaining insulin without HP-β-CD.

There are three disulfide bonds in insulin molecule, two of which link the two insulin chains. These disulfide bonds are critical to the biological activity of insulin, but they are sensitive to both reducing and oxidizing reagents. GSH can reduce and lyse the disulfide bond, therefore leading to the decomposition of insulin. A buffer containing GSH/GSSG was used in determining the stability of disulfide bonds. Different GSH(Glutathione(Reduced))/GSSG(Glutathione (Oxidized)) (mM/mM) solutions with GSH/GSSG at 0, 20/1, 40/1 and 60/1 were used to examine the stability of the disulfide bond. The degradation products were separated by pH 8.3 PAGE (polyacrylate gel electrophoresis), stained with Coomassie Brilliant Blue R-250. The PAGE results are shown in Figures 4A and B. As the GSH increased, insulin degradation increased accordingly, but as shown in the graphs, there were three degradation product bands when insulin was incubated without HP-β-CD, but only two bands when insulin was incubated with HP-β-CD. The main band (insulin) in Figure 4 (B) was much brighter, which indicated that more insulin remained. In addition, the HPLC results showed that the remaining insulin was more in the solution with HP-β-CD than in the solution without HP-β-CD. This indicated that HP-β-CD could increase the stability of disulfide bonds. It would be very interesting to know how HP-β-CD increases the stability of disulfide bonds. The mechanism will be the subject of further studies in our laboratory. Raman spectroscopy, which is a precise and accurate method for studying the character of disulfide bonds could be used.

### 2.5. Effects of HP-β-CD on the ^1^H-NMR spectrum of insulin

**Figure 5.**
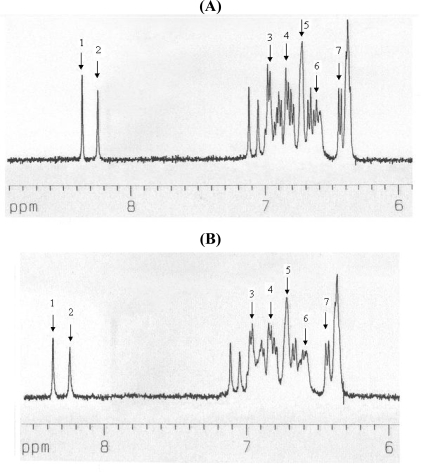
^1^H-NMR spectrum of aromatic region of insulin (4 mM) in the absence of HP-β-CD (A) and in the presence of HP-β-CD (120 mM) (B) in D_2_O containing 30% CD_3_COOD at room temperature.

The experiments above indicated that HP-β-CD could partially protect insulin from degradation. In an attempt to investigate the interaction of insulin with HP-β-CD, ^1^H-NMR spectroscopy in 30% CD_3_COOD was employed (D replaces H of CH_3_COOH). Figure 5 shows the effects of HP-β-CD on the ^1^H-NMR spectrum of the aromatic region of insulin. The first two peaks (the 1^st^ and the 2^nd^ arrows in Figure 5), well separated from the main aromatic region (6.2 – 7.0 ppm) were attributed to the C2 protons of the B5- and B10-histidines. The inclusion of the aromatic side chains within the cavity of HP-β-CD induced chemical shift displacements. The B5 and B10 histidines experienced a significant chemical shift, The changes in chemical shifts of A19 (the 3^rd^ arrow), B1 (the 4^th^ arrow), B26 (the 6^th^ arrow) and A14 (the 7^th^ arrow) were among the largest. The B24-phenylalanine (the 5^th^ arrow) is known to be directed towards the hydrophobic interior of the insulin molecule and its ring rotation is considerably restricted [18]; no noticeable change in the ^1^H-NMR signal of the B24-phenylalanine was noted by the addition of HP-β-CD, which may be ascribable to the difficulty of the CDs in gaining access to the folded side chain.

Improved insulin stability was observed due to the complexation with HP-β-CD. The ^1^H-NMR experiment indicated that complexation between HP-β-CD and insulin existed. Furthermore, the deamidation sites in insulin molecules might be included into the cone of cyclodextrin molecules, which increased the stabilization of the insulin., although there were some difficulties for HP-β-CD in approaching the side chain of insulin. Our results are consistent with those of Sajeesh *et al.* [11] who confirmed the formation of HP-β-CD and insulin complexes, using FTIR and fluorescence spectroscopic analysis.

## 3. Experimental Section

### 3.1. Materials

Insulin was purchased from Wanbang Biopharma Company, Xuzhou (nominal activity of insulin: 28I U/mg). HP-β-CD (MW: 1,380 Da, molar substitution 0.6) was obtained from Sigma Chemical Co. (USA). GSH and GSSG were purchased from Shanghai Biochemistry Pharma Company. Acetonitrile was HPLC grade, all other reagents used were of commercially available analytical grade.

### 3.2. HP-CD/Insulin complex preparation

HP-β-CD/insulin complex was prepared by mixing HP-β-CD and insulin (10:1, w/w), adding a small amount of purified water, and stirring for 2 hours at room temperature. The product is dried in a vacuum drying oven at room temperature.

### 3.3. Acid and base incubation

Acid insulin (and HP-β-CD/insulin complex) solution is prepared with 100 mL of 0.1 mol/L HCl solution (pH 1) and 10 mg insulin (110 mg HP-β-CD/insulin complex, in which the amount of insulin is equivalent to insulin only solution). Base insulin (and insulin complex) solution is prepared with 100 mL 0.1 mol/L NaOH solution (pH 13) and 10 mg insulin (110 mg HP-β-CD/insulin complex, in which the amount of insulin is equivalent to insulin only solution). All solutions were kept in 37 °C water for 16 hours. Samples were taken from the solutions every four hours and the concentrations immediately determined by HPLC.

### 3.4. High temperature incubation

Insulin (and HP-β-CD/insulin complex) solution of neutral pH is prepared with 100 mL pH 6.86 phosphate buffer solutions (PBS). Both solutions were kept in 55 °C water for 16 hours. Samples are taken from the solutions every four hours and the concentrations are immediately determined by HPLC.

### 3.5. Disulfide bond stability

Insulin was incubated in different GSH/GSSG (mM/mM) solutions at 0 °C for 12 h. The final concentration of insulin was 0.2 mmol/L. PAGE was then used to investigate the remaining insulin and its degradation products. PAGE was carried out according to Laemmli with minor modifications [19]. The running gel, stacking gel and electrode buffer were without sodium dodecyl sulfate (SDS). The running gel and stacking gel contained 15% and 4.5% acrylamide, respectively. After electrophoresis, the gels were subjected to stain with Coomassie Brilliant Blue R-250.

### 3.6. High Performance Liquid Chromatography (HPLC)

HPLC was carried out according to Todo *et al.* [20] with minor modifications. Analyses of remaining insulin were carried out by reverse phase HPLC with an isocratic system (Shimadzu Co., Kyoto, Japan) using the raw insulin bulk drug as the standard (28.0 U/mg). The HPLC system was composed of a pump (LC-10ADvp), diode array detector (SPD-M10Avp), column oven (CTO-10ASvp), and LC work station (CLASS-LC10). The mobile phase was a 72:28 mixture of 0.2 M sodium sulphate buffer (pH 3.0) and acetonitrile at a flow rate of 1.0 mL/min. The column was a Shodex Asahipak ODP-506D (4.6 mm × 150 mm, 5 um) (Showa Denko, Ltd., Tokyo) heated at room temperature. Ultraviolet absorption was measured at 214 nm.

### 3.7. Proton-nuclear magnetic resonance (^1^H-NMR) spectroscopy

The NMR spectra were measured according to Dotsikas *et al.* [13] with minor modifications. The ^1^H-NMR spectra of insulin (4 mM) in the absence and presence of HP-β-CD (120 mM) were obtained at 25 °C with a Bruker Analytik GmbH spectrometer (Avance DRX 400, Houston, USA), operating at 400.13 MHz, using 30% (v/v) CD_3_COOD in D_2_O as solvent. ^1^H-NMR chemical shifts were given in parts per million (ppm) relative to that of the HOD signal or CD_3_COOD signal with an accuracy of ±0.001. The ^1^H-NMR signals of aromatic region of insulin were assigned according to the reports of Hua and Weiss [20].

## 4. Conclusions

There has been a long history of research directed toward the development of novel routes of insulin delivery, but development of a proper non-invasive insulin delivery system remains a major challenge [22]. The stability of insulin has been extensively studied since insulin had first been used as a drug, early in the 1920s [17]. The stability of insulin is critical for its formulation, production and storage. Cyclodextrins have been used as stabilizers for many drugs and proteins [7,10,23–25]. Previous studies showed that CDs have many positive effects on the stabilization of insulin [13,14,26]. This work specifically studied the effect of HP-β-CD on insulin stability when treated with strong acid, strong base, and high temperature. Besides, effect of HP-β-CD on the stability of disulfide bonds were also been investigated. The results suggested that HP-β-CD could increase the stability of insulin under different conditions. Furthermore, the present study highlighted that HP-β-CD was also useful for the stability of the disulfide bonds in insulin. The stability of disulfide bonds is critical to the biological activity of insulin. Gould *et al.* [9] concluded that HP-β-CD was well tolerated in the animal species tested (rats, mice and dogs). Taking all this information into account, we conclude that HP-β-CD could offer positive and useful results in designing insulin preparations.

## Figures and Tables

**Table 1.** Effects of HP-β-CD on ^1^H NMR chemical shifts of insulin in 30% CD3COOD.

**No.**	**Side chain**	**Position**	**Insulin, chemical shift**	**With HP-β-CD (Δδ ppm)**
1	Histidine (B10)	C2	8.3679	−0.0102
2	Histidine (B5)	C2	8.2308	−0.0135
3	Tyrosine (A19)	C2 and 6	6.9688	−0.0704
4	Phenylalanine (B1)	C2 and 6	6.8336	−0.0517
5	Phenylalanine (B24)	C3 and 5	6.7211	−0.0016
6	Tyrosine (B26)	C2 and 6	6.6021	−0.0642
7	Tyrosine (A14)	C3 and 5	6.4613	−0.0581
